# Research Progress on the Pathogenesis, Diagnosis, and Drug Therapy of Alzheimer’s Disease

**DOI:** 10.3390/brainsci14060590

**Published:** 2024-06-09

**Authors:** Yixuan Yang, Lina Qiu

**Affiliations:** 1 College of Chemistry and Biological Engineering, University of Science and Technology Beijing, Beijing 100083, China; u202142497@xs.ustb.edu.cn; 2 Beijing Key Laboratory for Science and Application of Functional Molecular and Crystalline Materials, University of Science and Technology Beijing, Beijing 100083, China

**Keywords:** Alzheimer’s disease, amyloid-β, pathogenesis, diagnosis, drug therapy

## Abstract

As the population ages worldwide, Alzheimer’s disease (AD), the most prevalent kind of neurodegenerative disorder among older people, has become a significant factor affecting quality of life, public health, and economies. However, the exact pathogenesis of Alzheimer’s remains elusive, and existing highly recognized pathogenesis includes the amyloid cascade hypothesis, Tau neurofibrillary tangles hypothesis, and neuroinflammation hypothesis. The major diagnoses of Alzheimer’s disease include neuroimaging positron emission computed tomography, magnetic resonance imaging, and cerebrospinal fluid molecular diagnosis. The therapy of Alzheimer’s disease primarily relies on drugs, and the approved drugs on the market include acetylcholinesterase drugs, glutamate receptor antagonists, and amyloid-β monoclonal antibodies. Still, the existing drugs can only alleviate the symptoms of the disease and cannot completely reverse it. This review aims to summarize existing research results on Alzheimer’s disease pathogenesis, diagnosis, and drug therapy, with the objective of facilitating future research in this area.

## 1. Introduction

Alzheimer’s disease (AD) is a neurodegenerative disease divided into three stages: preclinical AD, mild cognitive impairment (MCI), and dementia [1]. The symptom of preclinical AD is cognitive impairment, and loss of independence is the primary feature differentiating dementia from MCI [2,3]. There are several other prevalent diseases in the elderly, such as Parkinson’s disease (PD) and amyotrophic lateral sclerosis (ALS). They share several common symptoms with AD. PD is a neurodegenerative disease. It is caused by mutations in some genes, including Pink1 (PARK6), Parkin (PARK2), DJ-1 (PARK7), and alpha-synuclein (PARK1). Its main symptoms are motor dysfunctions, but PD patients also suffer from comorbid non-motor symptoms, including cognitive decline, sleep disorder, and depression. Furthermore, its patterns of progression vary considerably across individuals [4,5,6]. ALS is a fatal neurodegenerative disease. It leads to muscle loss and axonal loss in the lateral spinal cord columns, but its pathophysiology remains incompletely understood [7,8]. 

AD is the single biggest cause of dementia among old people. As the population ages, the worldwide prevalence of dementia is expected to reach approximately 150 million by 2050, particularly in developing countries [9,10]. Furthermore, AD causes a substantial resource and economic burden on families and society. For example, it is estimated that in the United States in 2018, some family members spent more than USD 10,000 caring for AD patients, and the total cost for the nation was 277 billion [11].

Since the proposal of AD by Alois Alzheimer in 1906, through the pathological anatomy of a woman, the exact pathogenesis of AD had not yet been definitively identified. However, various hypotheses have been put forward, most notably the amyloid cascade hypothesis, Tau neurofibrillary tangles hypothesis, and neuroinflammation hypothesis [12,13,14,15,16]. With progressive research, it has also become evident that there is a certain level of interaction between these hypotheses [17,18].

The clinical diagnostic methods for AD include noninvasive neuroimaging positron emission computed tomography (PET), magnetic resonance imaging (MRI), and invasive cerebrospinal fluid (CSF) molecular diagnosis [19,20]. CSF molecular diagnosis has a high accuracy, but it is invasive. However, PET is expensive, and MRI cannot exactly distinguish AD from some other neurodegenerative diseases. In recent years, there has been a rise in using artificial intelligence to develop machine learning models to diagnose AD or distinguish between MCI and AD [21].

To date, there are still no clinical methods that can completely reverse AD, and the main therapy of AD is drugs. The early Food and Drug Administration (FDA)-approved AD drugs were cholinergic drugs, such as Tacrine, Donepezil, Rivastigmine, Galantamine, and the glutamate receptor antagonist Memantine [22,23]. Recently, the Aβ monoclonal antibodies Aducanumab and Lecanemab have been approved by the FDA for marketing through clinical trials [24,25]. Additionally, Sodium Oligomannate, the first drug targeting the brain-gut axis, has also been approved for marketing in China [23].

**Figure 1:**
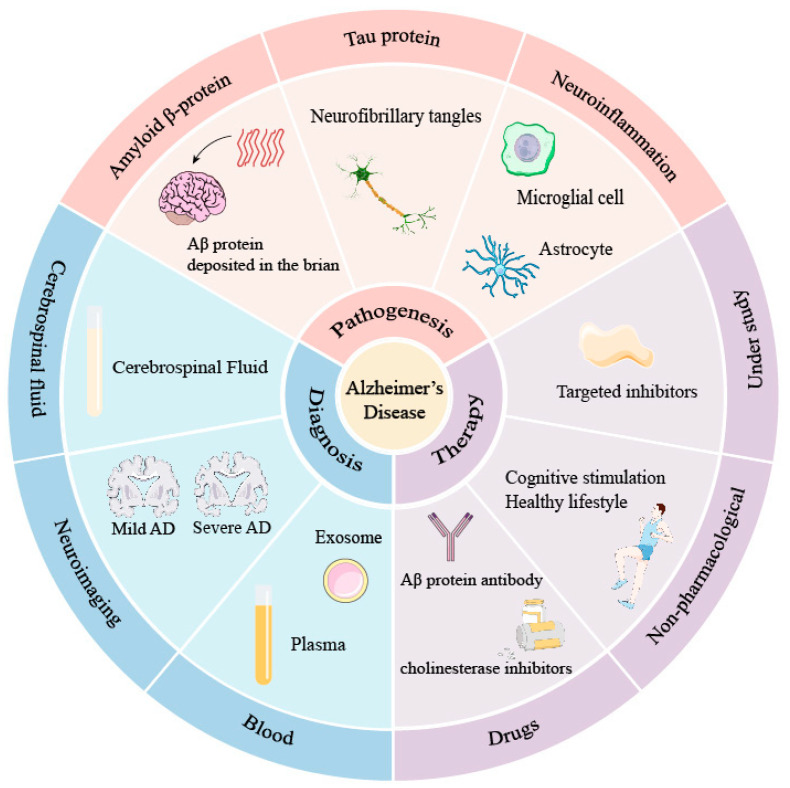
The pathogeneses, diagnoses, and therapies for AD.

In this review, we introduce the existing major pathogeneses of AD, including the amyloid cascade hypothesis, the Tau neurofibrillary tangles hypothesis, and the neuroinflammation hypothesis. Diagnostic methods for AD, including PET, MRI, and CSF molecular diagnosis, are discussed. We also list various FDA-approved drugs with their curative effects and side effects. By comparing existing pathogeneses, diagnoses, and drug therapies of AD, we aim to draw insights from previous research experiences and facilitate future studies into AD (Figure 1). 

## 2. Pathogenesis of Alzheimer’s Disease

### 2.1. Amyloid Cascade Hypothesis

The amyloid cascade hypothesis originated from observations made by researchers studying prion particles. They found that entities in brain slices from Creutzfeldt-Jakob disease were similar to plaques in AD brains described years ago [26]. The hypothesis suggests that the aggregation of amyloid-β (Aβ) plays a significant role in the development of AD.

**Figure 2:**
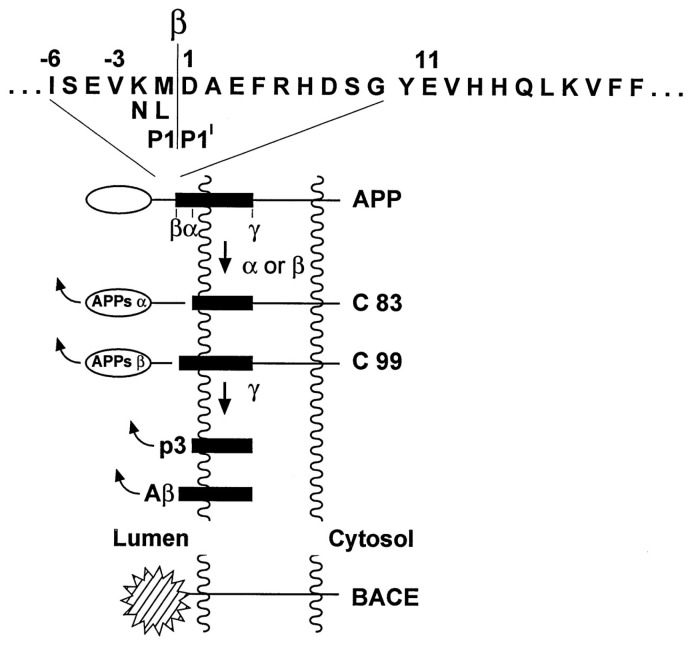
Schematic structure of APP and beta-site APP-cleaving enzyme (BACE) (not drawn to scale), showing proteolytic processing sites and cleavage products of APP [29].

Aβ is generated through hydrolysis of the amyloid protein precursor (APP) [27]. AβPP is a type-1 membrane protein expressed in various tissues, particularly in neuronal synapses. It plays an important role in the Aβ hypothesis. AβPP is composed of a transmembrane structural domain, a large extracellular glycosylated n-terminus, and a shorter cytoplasmic c-terminus [28]. There are two main pathways for the cleavage of AβPP in vivo (Figure 2 [29]): the main pathway is being cleaved by α-secretase to produce polypeptide chains with no aggregation [30]. The other is being cleaved by β-secretase to form CTF-β and then cleaved by γ-secretase to form aggregated Aβ of different lengths [31]. The panels related to AD typically contain Aβ_40_ and Aβ_42_ [10]. Aβ42 often has stronger aggregation [32] and neurotoxicity [33] than Aβ_40_. 

Aβ is toxic to neurons. Coordination compounds of Aβ and metal ions, such as Zn^2+^ and Cu^2+^, can release oxygen free radicals, causing oxidative damage to peripheral neurons [34]. Aβ can induce microglia to phagocytose synapses, resulting in the loss of synaptic function or synaptic disappearance [35]. Aβ can attach to the receptors on the surface of the neuronal membrane, causing Ca^2+^ to flow inward, increasing the Ca^2+^ concentration in neurons. This can result in mitochondrial dysfunction and apoptosis [36].

### 2.2. Tau Protein Hyperphosphorylation Hypothesis

The Tau protein hyperphosphorylation hypothesis suggests that neurofibrillary tangles, formed by hyperphosphorylation of Tau proteins, are important causes of AD. In 1988, Wischik et al. extracted Tau proteins from plaques in the brains of AD patients and first demonstrated that they might be implicated in dementia [37]. Tau protein, a type of microtubule protein, plays a critical role in promoting the assembly and stability of microtubules and the transport process of axons [38]. Tau protein is encoded by the MAPT gene on chromosome 17 [39] with a distinct primary structure, rarely with secondary and tertiary structures. Additionally, Tau protein is highly soluble and typically does not aggregate [40].

Tau protein has various modification pathways, with phosphorylation being the most significant. There are several phosphorylation sites in Tau, typically including threonine, tyrosine, and serine [41,42]. In a normal human brain, there are only 2–3 phosphorylation sites in Tau protein, and the phosphorylation and dephosphorylation of Tau can reach a dynamic balance. However, in the brains of AD patients, there can be as many as 40 phosphorylation sites, and there is a serious imbalance between phosphorylation and dephosphorylation, resulting in the formation of neurofibrillary tangles [43]. 

Studies have shown that reversible hyperphosphorylation of Tau proteins is a normal biological process during hibernation and sleep in animals. Reversible nonpathological phosphorylation of Tau depends on synergistic interactions between Tau kinases (such as Gsk3β, CdK5, etc.) and phosphatases (of which PP2A has the strongest catalytic role [44]) and alterations in the activity of either may lead to elevated Tau phosphorylation [45].

Later research has indicated that Tau hyperphosphorylation is associated with genetic mutations. Mutations in the MAPT gene on chromosome 17 increase the number of phosphorylation sites for Tau protein [46,47]. Tau neurofibrillary tangles are toxic to neurons. They lead to neuronal loss and affect microtubule assembly and stability [48,49]. Furthermore, they impede nutrient transport in microtubules, resulting in neuronal damage [50].

### 2.3. Inflammatory Hypothesis

In recent years, with the study of AD advancing, researchers have found that neurofibrillary tangles caused by Aβ and Tau proteins often trigger neuroinflammation in the brain. Neuroinflammation, in turn, promotes the aggregation of Aβ and Tau neurofibrillary tangles [51]. Consequently, the mechanism of neuroinflammation is now considered to be the third major mechanism of AD [52].

Neuroinflammation primarily involves microglia and astrocytes in the central nervous system. Some of the cytokines they produce can regulate their physiological activities, such as tumor necrosis factor-alpha (TNF-α), interleukins, etc. [53,54]. 

Microglia are “immunosurveillance” cells in the central nervous system. Under normal conditions, microglia remain in a resting state. When they are stimulated by an external stimulus, they can be activated to an M1 pro-inflammatory phenotype and an M2 anti-inflammatory phenotype to protect neuronal cells [55]. Normal microglia can clear Aβ and inhibit Tau aggregation [56,57,58]. However, when over-activated by Aβs, microglia release cytotoxic factors such as interleukins and TNF-α. This creates a prolonged neuroinflammatory environment, which is toxic to neuronal cells [59].

Astrocytes play a critical role in neuronal metabolism, especially glutamate uptake, and in inter-synaptic neural signaling [60]. Similar to microglia, astrocytes shift to an activated state in response to stimuli: an A1 neurotoxic state in neuroinflammatory environments and an A2 neuroprotective state in ischemic states [61]. Under normal circumstances, astrocytes can surround and remove Aβ, but excess Aβ or an inflammatory environment can activate astrocytes to the A1 form, leading them to promote Aβ production and Tau protein phosphorylation to form neurofibrillary tangles [62,63,64]. It has also been shown that activated microglia have a role in activating astrocytes [65].

### 2.4. Other Hypotheses

In addition to the first two dominant AD hypotheses and the neuroinflammatory hypothesis, several other hypotheses have been proposed. Furthermore, they offer a mutually reinforcing relationship with the three hypotheses mentioned above.

#### 2.4.1. Abnormal Mitochondrial Autophagy

Aβ and Tau neurofibrillary tangles lead to mitochondrial autophagy dysfunction, resulting in the accumulation of damaged mitochondria in the brain. They cannot be digested by the lysosome properly. Damaged mitochondria reduce ATP production capacity and enhance reactive oxidative species (ROS) production capacity, thus leading to oxidative stress due to excess ROS and a lack of energy sources for neurons. Ultimately, neuronal cells undergo apoptosis and produce positive feedback regulating the accumulation of AB and neurofibrillary tangles [66,67,68].

#### 2.4.2. Cholinergic Theory

Choline is essential for the synthesis of acetylcholine (ACh), an important neurotransmitter. Abnormal signaling and function of the cholinergic system leads to cognitive deficits [69]. Studies have shown that cholinergic lesions in AD mainly occur presynaptic, resulting in dysfunction of muscarinic-type ACh receptors on presynaptic membranes, loss of nicotinic-type ACh receptors on postsynaptic membranes, and abnormalities of ACh transmission in nerve cells [70]. Cholinergic systemic heterogeneity also promotes the accumulation of Aβ and the formation of neurofibrillary tangles [71].

#### 2.4.3. Insulin Resistance

Studies have shown that one of the common symptoms of both AD and type-2 diabetes is insulin resistance [72,73,74]. In addition to its role in glucose metabolism in the brain, insulin is also involved in signaling between neurons, promoting AβPP synthesis, and the phosphorylation process of Gskβ [75]. A lack of insulin leads to the deposition of Aβ and hyperphosphorylation of Tau [68,76].

#### 2.4.4. Abnormal Gut Microflora

The gut microflora maintains bi-directional interactions with key parts of the central nervous system and the immune system through direct and indirect pathways [77]. As gut microbial abundance changes due to the aging process or daily diet, the microbial balance can be disrupted. The disruption triggers neuroinflammation through the brain-gut axis, promotes the travel and deposition of Aβ and Tau proteins, and exacerbates insulin resistance. These effects all worsen the AD condition [78,79,80,81].

#### 2.4.5. Presenilin Hypothesis

Genetic mutations in presenilin 1 (PSEN1), presenilin 2 (PSEN2), and the amyloid precursor protein (APP) are major causes of familial AD(FAD) and early-onset AD(<65 years old) [82]. PSEN1 and PSEN2 are essential components of the γ-secretase complex and can impair the cleavage of APP by γ-secretase. Mutations in PSEN1 and PSEN2 can increase the production of Aβ_40_ and Aβ_42_ [83]. PSEN1 mutations are more pathogenic than PSEN2 due to their higher frequency of mutations, and the onset age of carriers of PSEN1 mutations can be as early as 28 [84,85].

#### 2.4.6. Calcium Hypothesis

Calcium (Ca^2+^) is a requisite second messenger in all living organisms, and it regulates several physiological activities of neurons, including growth and differentiation, synaptic plasticity, learning and memory, necrosis, apoptosis, and degeneration [86,87]. Recent evidence indicates that Ca^2+^ dyshomeostasis is closely interrelated with AD. Mutations in PSEN1 and PSEN2 can interact with the inositol 1,4,5-trisphosphate receptor (InsP_3_R) Ca^2+^ release channel. These interactions exaggerate the influx of Ca^2+^ and cause Ca^2+^ dyshomeostasis [88]. Mitochondria plays an important role in absorbing Ca^2+^. Studies have shown that there is a mitochondrial Ca^2+^ dysregulation in AD, and this can lead to the production of ROS, inhibition of ATP synthesis, and activation of caspases and apoptosis [89,90,91]. Furthermore, Aβ can induce mitochondrial Ca^2+^ overload [92].

#### 2.4.7. Oxidative Stress

Studies have shown that elevated markers for oxidative stress precede Aβ deposition and Tau neurofibrillary tangles [93]. ROS are normally maintained at a low level in vivo and act as signaling molecules to mediate several signaling pathways. However, excessive ROS can lead to oxidative stress and be toxic to cells. This is especially damaging to neurons due to their high demands for oxygen [94,95]. It has been found that mutations of mitochondrial DNA, abnormal mitochondrial autophagy, and the accumulation of Aβ and Tau can increase the production of ROS [66,96,97]. In turn, oxidative stress can also increase the production of Aβ and the phosphorylation of Tau, which aggravates the AD condition [98,99].

## 3. Diagnosis of Alzheimer’s Disease

### 3.1. Cerebrospinal Fluid Molecular Diagnosis

The massive deposition of Aβ in the brain, neurofibrillary tangles formed by abnormal deposition of Tau proteins, and inflammatory factors are listed in the previous section as important contributors to the onset of AD. Likewise, these substances can be used as markers for early AD screening. The three most recognized CSF markers in current studies are Aβ_42_, t-Tau protein (total Tau protein), and p-Tau (phosphorylated Tau) [100]. 

CSF molecular diagnosis is invasive. It needs to perform a lumbar puncture on the patient and collect a CSF specimen of sufficient sample volume, as it affects the composition of the CSF [101]. Numerous experiments have shown that AD patients have reduced levels of Aβ_42_ and increased levels of t-Tau and p-Tau in their CSF [102]. It has been shown that abnormalities of the Aβ_42_ protein in CSF can lead to an earlier diagnosis of AD than PET imaging of Aβ [103]. However, it is also controversial that Aβ and Tau positivity do not fully confirm the diagnosis of AD. There are no defined criteria for the diagnosis of AD for these three markers due to differences in conditions and samples between laboratories [104,105]. Moreover, some studies have demonstrated that the ratio of Aβ_42_ to Aβ_40_ characterizes the results more accurately than Aβ_42_ alone [106,107,108].

### 3.2. PET Neuroimaging Diagnosis

**Figure 3:**
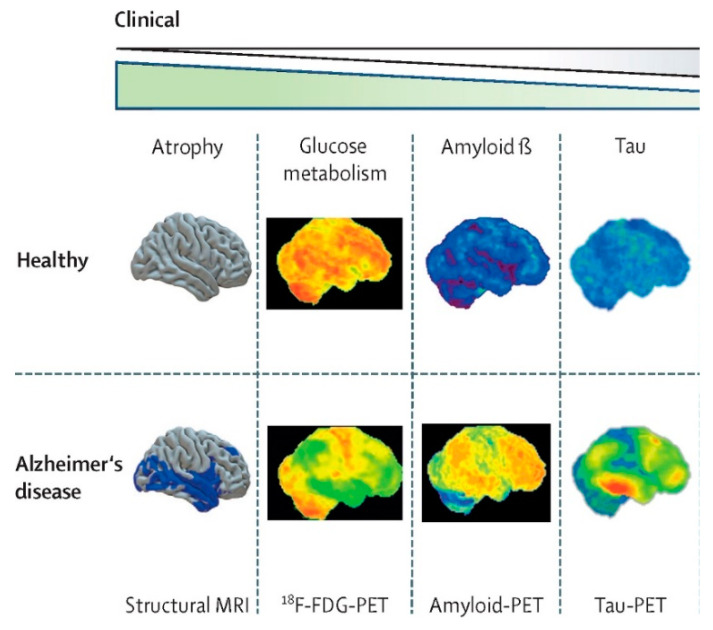
Neuroimaging obtained with structural MRI or PET using different radiotracers [109].

The structure of the patient’s brain can be observed through PET imaging, including its shape, size, and depositions (Figure 3 [109]). It can be used to differentiate between AD and MCI as well as to predict the process of transformation from MCI to AD. 

There are two main PET imaging types: ^18^F-flurodeoxyglucose positron emission computed tomography (^18^F-FDG-PET) and amyloid PET. ^18^F-FDG-PET combines ^18^F-FDG with PET. As it is known that glucose is the fundamental source in the brain, assessing glucose consumption in certain regions can indicate neuronal dysfunctions. ^18^F-FDG-PET is proven to be 12% more precise in predicting the process of transformation from MCI to AD compared to MRI and CSF molecular diagnosis [110]. Amyloid PET possesses a significantly higher sensitivity in predicting MCI progression to AD, reaching 93%, much higher than ^18^F-FDG-PET [111]. Experiments have shown that detecting Aβ and Tau proteins alone is similar to detecting them together. Detecting them individually can reduce the cost of diagnosis for hospitals and patients [112].

### 3.3. MRI Neuroimaging Diagnosis

MRI is more commonly used to distinguish between AD and MCI [113]. There are two types of MRI: structural MRI and functional MRI. Structural MRI assesses the atrophy of critical brain regions and cortical thickness [114]. Functional MRI studies activation or functional connectivity and proton magnetic resonance spectroscopy for the N-acetylaspartate (NAA)/creatine ratio in specific areas [115,116]. It has been proven that medial temporal atrophy, particularly hippocampal atrophy, is the best MRI marker [117,118]. However, some other neurodegenerative diseases exhibit similar atrophy to AD [1].

### 3.4. Blood Tests

#### 3.4.1. Plasma Testing

Neuronal cells can secrete vesicle-like exosomes for the transport of metabolic wastes and signal transduction among neurons [119]. Exosomes play an important role in the synthesis and transport of Aβ and Tau proteins [58,120,121]. By detecting elevated levels of Aβ and Tau proteins in exosomes, AD can be detected ten years earlier than clinical diagnosis with an accuracy rate of 96% [120,122]. Exosomes contain several miRNAs, and their relative content changes when AD occurs, so the extraction and analysis of miRNAs is another way to detect AD [123]. 

Many previous studies indicated that there was no difference between plasma Aβ of AD patients and those of healthy controls [124,125,126], but some studies have shown that there is a decline of Aβ_42_ and an increase of Aβ_40_ [127,128]. The results correlated with CSF tests and PET tests and indicated that plasma Aβ biomarkers are strongly linked with the Aβ status of the central nervous system but less affected by the Aβ known to be produced in peripheral tissues [129,130,131]. Recent studies have indicated that there is Aβ misfolding in the plasma of AD patients [132]. Additionally, the plasma Aβ_42_/Aβ_40_ decline can be used to predict the risk of AD, and it is highly consistent with the result of PET tests [133]. 

Recent studies have shown that the accuracy of plasma p-Tau testing has been comparable to CSF molecular diagnosis, and the range of testing includes preclinical AD [134].

#### 3.4.2. Blood–Brain Barrier Testing

It has been shown that the deposition of the APOEε4 gene, rather than Aβ protein and Tau protein, can accelerate the disruption of the blood–brain barrier, which is associated with early cognitive impairment [135,136].

#### 3.4.3. Serum Testing

Some studies have shown that there is a significant decrease in brain-derived neurotrophic factors in the serum of AD patients compared with that of healthy people. The measurement of brain-derived neurotrophic factor content is expected to be used as a test indicator for AD [137,138]. A study led by King’s College London and the University of Oxford extracted serum from patients with MCI who converted to AD at a later stage and from MCI patients who remained cognitively stable at a later stage. Then, they were applied to treat hippocampal cells and tested the effect of the serum on the process of hippocampal genesis in vitro. By drawing comparisons with healthy controls, the result can make predictions about the conversion of MCI to AD, and it is expected to predict the onset of AD up to 3.5 years in advance. Additionally, it is more accurate and comprehensive than ordinary proteomics tests with the diagnosis of all proteins in the serum. However, this test is less accurate than CSF molecular diagnosis [139].

### 3.5. Artificial Intelligence Diagnosis

With the rapid development of artificial intelligence, diagnostic methods related to machine learning and prediction computer algorithms are gradually emerging (Table 1). Since the atrophy of the hippocampal region of the brain is significantly associated with the onset of AD and the degree of dementia, the latest research aims to build a deep-learning model based on MRI and PET to predict AD-level images [140]. A recent study used brain MRI image segmentation techniques in particular. They constructed a deep-learning model that only directed on the hippocampal region of the brain. The study utilized two datasets, Kaggle and OASIS, to build a model. Training the model to extract the hippocampal region from brain MRI images in the OASIS dataset, the Kaggle dataset served as the testing set after selecting the best model. The final results showed that the method can reach the simplification of existing algorithms while guaranteeing the accuracy of predicting AD [21].

## 4. Therapy of Alzheimer’s Disease

### 4.1. Acetylcholinesterase Inhibitors

**Figure 4:**
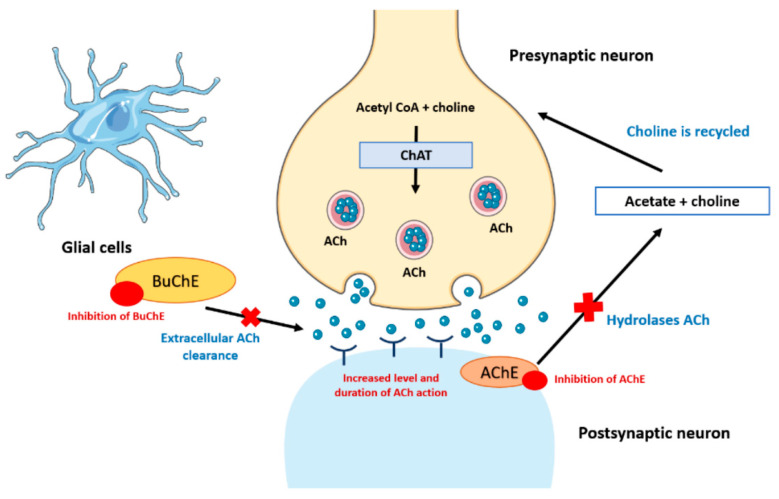
Cholinergic hypothesis in pathogenesis and treatment of AD [145].

Tacrine: Tacrine is the earliest anti-AD drug. It is an acetylcholinesterase (AChE) inhibitor and reduces its catabolism of ACh. It enhances cholinergic effects to maintain neuronal excitability and ensures normal memory and cognitive functions [143,144] (Figure 4 [145]). Tacrine used to be the most effective AD drug, but it is no longer used due to its strong hepatotoxicity and excessive adverse effects [146]. In recent years, research on Tacrine derivatives has emerged, aiming to maintain Tacrine’s efficacy while minimizing its side effects on humans [147]. 

Donepezil: Donepezil is a second-generation AChE inhibitor [148]. In addition to inhibiting cholinesterase, it acts at the molecular and cellular levels at almost all stages of AD pathogenesis, including inhibiting aspects of glutamate-induced excitotoxicity, reducing early expression of inflammatory cytokines, and reducing oxidative stress induction [149]. Donepezil has various side effects, such as insomnia, nausea, loss of appetite, muscle cramps, and muscle weakness. Side effects worsen with increasing doses [150]. Donepezil is an approved drug for all stages of AD [151]. 

Rivastigmine: Rivastigmine, also known as carboplatin tartrate, is a second-generation AChE inhibitor with a mechanism similar to that of Tacrine and Donepezil. Rivastigmine prefers to bind to G1-type AChE, which plays a major role in synaptic cholinergic hydrolysis [152]. Its inhibition of cholinesterase can last up to 10 h, which is much higher than that of Tacrine, Donepezil, and Galantamine [153]. Rivastigmine is selective for the central nervous system and causes less damage to the peripheral nervous system. Rivastigmine is mainly used for the treatment of mild and moderate AD, with its side effects focusing on the gastrointestinal area [154]. 

Galantamine: Galantamine is a second-generation AChE inhibitor. It is selective for the central nervous system and typically binds to nicotinic cholinergic receptors [152]. However, resistance to Galantamine may occur with increasing doses, as well as side effects such as convulsions, severe nausea, stomach cramps, vomiting, irregular breathing, confusion, and muscle weakness [149]. 

All drugs above are FDA-approved AD drugs for clinical use [155]. Researchers have tried to develop their derivatives or combine several of them to enhance their effectiveness [152]. 

### 4.2. Glutamate Receptor Antagonists

Memantine is another AD drug approved by the FDA. Excessive accumulation of glutamate in the synaptic gap continuously acts on NMDA receptors, triggering an inward flow of Ca^2+^ and sustained neuronal excitation, resulting in neuronal apoptosis [156]. Memantine is a non-competitive NMDA receptor antagonist that impedes the binding of glutamate to NMDA receptors by lowering glutamate levels, thus decreasing Ca^2+^ inward flow and maintaining the normal physiological activity of neurons. Clinical trials have shown that Memantine can effectively slow down the process of cognitive decline [157], but its efficacy is not obvious for mild AD. Memantine is more suitable for the treatment of moderate AD [31]. The most common adverse effects of Memantine are dizziness, headache, and confusion. A small percentage of patients may experience agitation [158].

### 4.3. Aβ Monoclonal Antibody

Aducanumab Monoclonal Antibody: Aducanumab is a humanized antibody targeting Aβ and is the first anti-Aβ drug approved by the FDA. Aducanumab barely interacts with Aβ monomers but binds highly selectively to aggregated Aβ by recognizing the N-terminal residues of Aβ [159]. Its mechanism includes activating microglia to phagocytose Aβ through specific binding to Aβ. Aducanumab can originally inhibit Aβ by impeding the formation of Aβ oligomer on the surface of primary fibers [160]. Numerous experiments have demonstrated that Aducanumab can dose-dependently remove Aβ from the human brain. However, Aducanumab has not been demonstrated to have a significant effect on the alleviation of AD symptoms in phase III trials, so there has been a controversy over the marketing of Aducanumab [161]. In terms of safety, trials have shown high-dose Aducanumab to have a 40% chance of side effects, such as cerebral edema, sulcus effusion, and cerebral hemorrhage. The incidence rate increases with dose, and it is also higher in ApoEϵ4 carriers [162]. Recent studies have shown that using MRI to temporarily open the blood–brain barrier before taking Aducanumab can significantly reduce Aβ levels [163]. 

**Figure 5:**
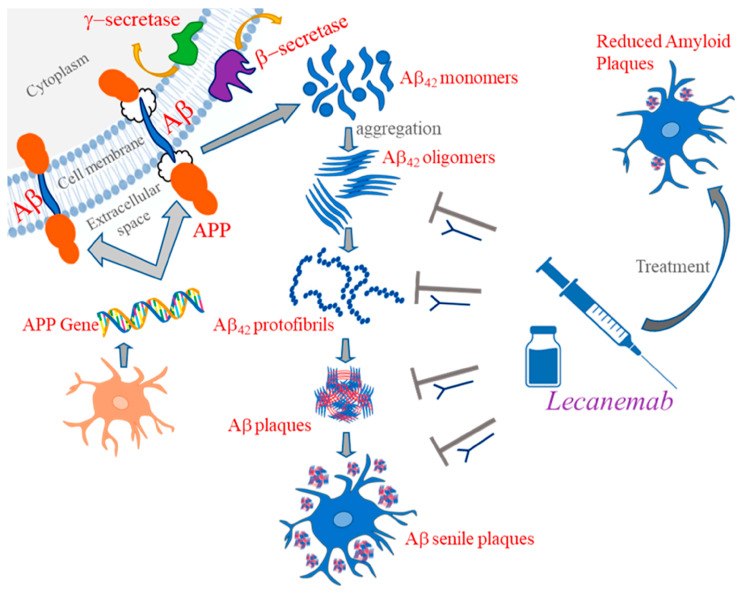
Mechanism of action of Lecanemab [164].

Lecanemab Monoclonal Antibody: In early 2023, the FDA formally approved a second drug targeting Aβ called Lecanemab. Lecanemab is a humanized lgG1 monoclonal antibody [25]. Opposed to the controversial phase III clinical trial of Aducanumab, the phase III trial of Lecanemab clearly demonstrated its effectiveness in relieving cognitive decline. Lecanemab binds tiny protofibrils with 100 times the affinity of Aducanumab and big protofibrils with 25 times the affinity, with lower binding affinity for monomers (Figure 5) [164]. Additionally, subsequent trials have proved that Lecanemab is effective in prolonging the MCI period to slow the progression of AD [165]. However, Lecanemab can cause minor cerebral hemorrhage and rare macrohemorrhage when removing Aβ [166]. 

### 4.4. The Drug Targeting the Brain-Gut Axis

Sodium Oligomannate (GV-971): Sodium Oligomannate is extracted from seaweed and is usually taken orally in capsules [167]. It has completed the phase III clinical trial and was approved for marketing in China [168]. It is the first oligosaccharide anti-AD drug targeting the cerebral-intestinal axis in the world [169]. Sodium Oligomannate can regulate the balance of intestinal flora. This inhibits the activation of inflammatory cells in the brain and helps clear the aggregation of Aβ as well as Tau in the brain, therefore alleviating mild cognitive impairment [168]. Compared to several FDA-approved drugs, Sodium Oligomannate has fewer side effects, but it is also relatively less effective (Table 2).

### 4.5. Other Drugs under Study

There are many drugs targeting other targets being researched and developed (Table 3). Drugs targeting the process of Aβ deposition and Tau protein phosphorylation remain at the forefront of research and development [170], while others target the process of inflammatory factor production by glial cells [171], the antibodies of β-secretase [172], and inhibitors of the Ca^2+^ channel, which regulates the oxidative stress response [173], etc.

### 4.6. Nonpharmacological Therapy

There are also various nonpharmacological therapies to alleviate the symptoms of AD. When the symptoms are mild, cognitive stimulation therapy can be used to help the AD patient relive sounds, faces, numbers, and other areas involved in daily life. It is a way to mentally stimulate the patient [182,183]. Exercise can improve blood flow and metabolic rate in the brain [184].

## 5. Summary and Prospects

For decades, there have been some advances in the research into the pathogenesis, diagnosis, and therapy of AD. However, the research on each remains incomplete, leaving many uncertainties about AD.

In terms of pathogenesis, although the exact pathogenesis of AD remains undetermined, several hypotheses have been supported. The three major hypotheses are the amyloid cascade hypothesis, Tau neurofibrillary tangles hypothesis, and neuroinflammation hypothesis. It has been proved that abnormal mitochondrial autophagy, insulin resistance, abnormal gut microflora, mutations of PSEN, calcium dyshomeostasis, and oxidative stress can also lead to AD. In recent years, researchers have found that a single hypothesis cannot fully explain the pathogenesis of AD, and there is a certain interaction between the various mechanisms. For example, abnormal mitochondrial autophagy can aggravate the deposition of Aβ and Tau neurofibrillary tangles.

The diagnosis methods for AD are mainly categorized into CSF molecular diagnosis, neuroimaging testing, and emerging blood tests. Through the efforts of researchers, the accuracy of blood tests has now reached the level of CSF molecular diagnosis, which is cheaper than neuroimaging testing. Additionally, the diagnostic markers are becoming more diverse, not only limited to Aβ and Tau protein, but also attempting to detect the relative content of miRNA, the plasma Aβ, blood–brain barrier, and cerebral blood flow.

In terms of therapy, there is still no therapy that can effectively reverse AD. Approved drugs are mainly cholinergic drugs, but all of them have certain side effects. Recently, monoclonal antibodies targeting the Aβ have been approved for marketing after rounds of clinical trials. Many experiments have confirmed the possible existence of some new targets, and the derivatives of existing drugs or the combinations of several drugs are also being put into experiments.

Future research on AD still needs to be vigorously pursued. For pathogenesis, future research should focus on the interactions between existing pathogeneses and follow the existing hypotheses to find other possible pathogeneses to cure the disease. For diagnosis, there are two challenges: one is how to diagnose AD at an early stage, and the other is the distinction between AD and MCI. In the future, we can find more possible biomarkers through the existing pathogeneses of AD or compare the blood components of the AD model with the healthy model to find out other abnormal indicators of the AD model. In addition, it is also necessary to popularize the importance of early screening for AD, as most of the existing drugs for AD are aimed at the early and middle stages of AD. To increase awareness for early screening, the possible symptoms of the latent stage of AD should be popularized to society as well. For drug therapy, due to the uncertainty of the pathogenesis and the ethical issues in experimental research, the process of drug research has been relatively slow, and the efficacy is relatively insufficient. However, we can reduce the risk of AD as it has been pointed out that AD is closely related to daily behavioral habits such as sleep, diet, smoking, and alcohol. Therefore, it is important to practice healthy living habits such as exercising to maintain good physical health to prevent AD [4]. Moreover, brain-computer interface (BCI) technology has gradually emerged in recent years. There have been studies about using BCI to help stroke people operate a mechanical arm [185,186]. Several studies have suggested that using BCI technology can control objects through the consciousness of animals, such as monkeys and pigs. Furthermore, BCI technology has already been applied in studies of PD treatments and ALS treatments [187]. In early 2024, Neuralink completed the first BCI implantation in the human brain, and the patient recovered well. We think BCI technology has a potential development in AD treatment and assists in the movement of AD patients.

## Figures and Tables

**Table 1:** Diagnostic methods and their makers, advantages, and disadvantages.

Method	Marker	Advantage	Disadvantage	References
CSF molecular diagnosis	Aβ42 T-Tau P-Tau	accurate	invasive, high rate of misclassification	[102,108,141]
PET	glucose metabolism Aβ, Tau protein	noninvasive, sensitive	expensive, confused with other diseases	[111,112]
MRI	medial temporal atrophy	noninvasive	expensive, confused with other diseases	[1,142]
blood tests	plasma exosomes plasma Aβ40, Aβ42, p-Tau serum blood–brain barrier	minimally invasive, diversity of markers	relatively less accurate	[120,122,129,139]
artificial intelligence diagnosis	a deep-learning model based on MRI and PET	efficient	immaturity of technology	[140]

**Table 2:** Approved AD drugs and their side effects.

Name of Drug	Type of Drug	Side Effect	Approved by	References
Tacrine	acetylcholinesterase inhibitor	strong hepatotoxicity	FDA	[146]
Donepezil	acetylcholinesterase inhibitor	insomnia, nausea, loss of appetite, muscle cramps, muscle weakness	FDA	[150]
Rivastigmine	acetylcholinesterase inhibitor	Relatively low side effects focusing on the gastrointestinal area	FDA	[154]
Galantamine	acetylcholinesterase inhibitor	convulsions, severe nausea, stomach cramps, vomiting, irregular breathing, confusion, muscle weakness	FDA	[149]
Memantine	glutamate receptor antagonist	dizziness, headache, and confusion	FDA	[158]
Aducanumab	Aβ monoclonal antibody	cerebral edema, sulcus effusion cerebral hemorrhage	FDA	[160]
Lecanemab	Aβ monoclonal antibody	minor cerebral hemorrhage rare macrohemorrhage	FDA	[166]
Sodium Oligomannate	the drug targeting the brain-gut axis	Relatively low side effects	China-FDA	[168,169]

**Table 3:** Other drugs under study.

	Mechanism of Action	Phase of Clinical Trials	References
(1)	Aβ aggregation inhibitors	in phase 2	[174]
(2)	α-secretase modulators	most are in phase 2	[175,176]
(3)	β-secretase inhibitors	most are in phase 1 and 2, the farthest and is in phase 2/3	[171,177]
(4)	γ-secretase inhibitors	failed in phase 3	[178]
(5)	inhibitors of Tau hyperphosphorylation	failed in phase 2	[31]
(6)	Tau protein aggregation inhibitors	failed in phase 3	[179]
(7)	drugs that enhance Tau clearance	in phase 1	[179,180]
(8)	intranasal insulin	in phase 2	[181]
(9)	TREM2-activating antibodies	preclinical	[171]
(10)	Ca^2+^ channel inhibitors	preclinical	[173]
